# Carrier Screening: Past, Present, and Future

**DOI:** 10.3390/jcm3031033

**Published:** 2014-09-15

**Authors:** Komal Bajaj, Susan J. Gross

**Affiliations:** 1 Department of Obstetrics & Gynecology Women’s Health, Albert Einstein College of Medicine, 1400 Pelham Pkwy South, 1BS16, Bronx, NY 10461, USA; 2 Department of Obstetrics & Gynecology Women’s Health, Albert Einstein College of Medicine, 1300 Morris Park Avenue, Block Building (Rm 623), Bronx, NY 10461, USA; E-Mail: susan.gross@einstein.yu.edu

**Keywords:** genetic screening, prenatal care, genomic medicine

## Abstract

To date, preconceptual and prenatal patients have been offered gene-by-gene, disorder-by-disorder carrier screening. Newer techniques allow screening of many disorders at one time. The goal of this review is to provide an overview of the current practice and future direction of carrier screening within the preconceptual/prenatal setting.

## 1. Introduction

Carrier screening has been a part of clinical practice for several decades. Recent advances in molecular technology have catapulted options for carrier screening from a gene-by-gene evaluation to panels spanning many disorders at one time. For the purposes of this article, we will review the past, present, and future of genetic carrier screening as it relates to the preconceptual and prenatal patient.

While the process of carrier screening has always entailed the identification of mutations within individuals who are pregnant or considering pregnancy, the actual goal of such programs is the prevention of genetic disease in subsequent generations. Of the over 7000 diseases thought to exhibit Mendelian inheritance, more than 15% have an established molecular etiology that is recessive in nature [1]. For autosomal recessive conditions, carriers are asymptomatic but children of couples who are both carriers have a 25% chance of being affected by the condition. Carrier screening empowers such couples with respect to their family planning options and may provide reassurance to those individuals who are screen negative. In 1968, Wilson and Junger outlined criteria to guide the selection of conditions suitable for screening. These criteria, listed in Table 1, were updated by the World Health Organization in 2008 to reflect changes in technology and understanding of disease:

**Table 1:** Updated Wilson-Junger screening criteria [2].

Wilson and Jungner Classic Screening Criteria	Synthesis of Emerging Screening Criteria Proposed over the Past 40 Years
The condition sought should be an important health problem.	The screening program should respond to a recognized need.
There should be an accepted treatment for patients with recognized disease.	The objectives of screening should be defined at the outset.
Facilities for diagnosis and treatment should be available.	There should be a defined target population.
There should be a recognizable latent or early symptomatic stage.	There should be scientific evidence of screening program effectiveness.
There should be a suitable test or examination.	The program should integrate education, testing, clinical services and program management.
The test should be acceptable to the population.	There should be quality assurance, with mechanisms to minimize potential risks of screening.
The natural history of the condition, including development from latent to declared disease, should be adequately understood.	The program should ensure informed choice, confidentiality and respect for autonomy.
There should be an agreed policy on whom to treat as patients.	The program should promote equity and access to screening for the entire target population.
The cost of case-finding (including diagnosis and treatment of patients diagnosed) should be economically balanced in relation to possible expenditure on medical care as a whole.	Program evaluation should be planned from the outset.
Case-finding should be a continuing process and not a “once and for all” project.	The overall benefits of screening should outweigh the harm.

The identification of carriers gives patients who are either contemplating a pregnancy or those that are early along in pregnancy to make decisions regarding prenatal diagnosis. After thorough genetic counseling, a pregnant patient may opt for prenatal diagnosis by either chorionic villus sampling or amniocentesis. Conversely, the pregnant patient may decline invasive testing and instead opt for expectant management until birth. A preconceptual patient may choose to capitalize upon advances in assisted reproduction technologies such as *in vitro* fertilization followed by preimplantation genetic diagnosis to minimize the risk of an affected fetus.

Genetic carrier screening has traditionally been offered to patients based on their family history or ethnic background, where individuals deemed to be at higher-risk of being a carrier are screening for a limited number of mutations that account for the majority of the disease in question. Few diseases have been considered by professional organizations or government agencies to be common and/or significant enough to recommend that all individuals, regardless of background, be offered screening. Within the United States, cystic fibrosis serves an example of such a “pan-ethnic” screening approach in the preconceptual/prenatal patient. Globally, hemoglobinopathies and thalassemias have been the focus of many population-wide screening programs and is recommended by the American College of Obstetrics and Gynecology (ACOG) in the United States for pregnant women from high risk backgrounds [3].

Conditions that have traditionally been suitable for more focused population screening are those for which specific mutations are found in individuals of a defined ancestral background. These mutations are usually the result of a “founder effect”—a mutation present in a few individuals in a small population is transmitted to descendants, resulting in an increased incidence of particular autosomal recessive disorders caused by these mutations. A well-known example of this are a group of autosomal recessive disorders, including Tay-Sachs Disease (TSD), in the Eastern European (Ashkenazi) Jewish population. Known as “Jewish Genetic Diseases”, this panel of disorders continues to increase as mutations specific to this population are identified.

The story of how screening for Cystic Fibrosis and Tay-Sachs Disease has evolved demonstrates how the approach to screening, both from a counseling and testing perspective, has changed over time within the United States. Cystic Fibrosis is the most common autosomal recessive disorder impacting the Caucasian population. Due an abnormality in chloride channels, classic features of CF include pulmonary and digestive dysfunction. CFTR, the gene associated with cystic fibrosis, was identified in 1989. ΔF508, a deletion of phenylalanine at position 508, is the most common mutation and accounts for approximately two-thirds of cystic fibrosis cases worldwide. In 1997, the National Institutes of Health Consensus Development (NIHCD) Conference on Genetic Testing for Cystic Fibrosis recommended that genetic screening for cystic fibrosis (CF) be offered to individuals with a family history of CF, to partners of individuals with CF, to couples considering a pregnancy and to individuals seeking prenatal care [4]. Shortly thereafter, ACOG, as well as the American College of Medical Genetics and Genomics (ACMG), introduced guidelines in 2001, recommending that screening be offered to “high-risk” ethnic groups and considered in others. These guidelines were subsequently revised to recommend offering screening to all preconceptual/prenatal patients, regarding of ethnic background [5,6]. ACOG and ACMG recommended that patients opting for screening undergo evaluation for the 23 most common mutations associated with CF. The sensitivity of CF screening using a 23-mutation panel varies based on ethnic background and is most efficacious in non-Hispanic white and Ashkenazi Jewish populations. Patients need to be aware that a “negative” screening does not completely exclude the possibility of being a carrier for CF. Table 2 highlights CF detection rates and the post-test carrier risk, based on a 23-mutation screening panel. Now that almost two thousand disease-causing mutations have been identified and there are new approaches for understanding genotype/phenotype correlations, maintaining such a limited panel is coming under review. While it is possible that professional organizations may recommend increasing this mutation panel in the not too distant future, in practice, larger panels of CF mutations are already readily available within the U.S. suggesting that in this era of brisk technological advances, clinical reality may antecede professional guidance.

**Table 2:** Cystic fibrosis detection rates and carrier risk before and after testing [5].

Racial or Ethnic Group	Detection Rate (%)	Carrier Risk before Testing	Approximate Carrier Risk after Negative Test Result
Ashkenazi Jewish	94	1/24	1/380
Non-Hispanic White	88	1/25	1/200
Hispanic White	72	1/58	1/200
African American	64	1/61	1/170
Asian American	49	1/94	1/180

Unlike Cystic Fibrosis, which is now a pan-ethnic screen, Tay-Sachs Disease (TSD) screening has been targeted towards specific populations, including Ashkenazi Jews (AJ), French-Canadians and individuals of Cajun descent. The carrier frequency in individuals of AJ descent is 1/30 whereas the risk of being a carrier approaches 1/300 in the general population [7]. TSD is a lysosomal storage disease due to deficiency in Hexosamidase A, which results in progressive neurologic degeneration. Screening for TSD began in the 1970s utilizing biochemical evaluation of Hexosamidase A activity to identify carriers. Subsequently, three mutations identified in the HEXA gene were reported to account for 92%–98% of all TSD carriers in the AJ population, whereas 100+ disease-causing mutations have been identified in non-Jewish carriers [8]. Because three mutations account for the majority of carriers within the AJ population, molecular screening is highly sensitive within this population, though a strategy that uses only molecular screening without HexA activity assay would miss up to 10% of carriers within the Jewish population [9]. Widespread implementation of TSD carrier screening has significantly decreased the incidence of disease in the AJ population. In fact, the majority of babies now born with TSD are born to non-Jewish individuals. Tay-Sachs Disease, while often regarded as the prototype of a focused population test, may become a model of “high risk” tests moving into broad based screening algorithms.

Testing for hemoglobinopathies, however, still remains targeted to those at “high risk”, which is essentially everyone not of Caucasian background. Hemoglobin is composed of both alpha and beta globin chains. The reduction or complete absence of these chains results in alpha or beta thalassemia, respectively. Thalassemias comprise some of the most common autosomal recessive conditions that impact many different populations world-wide, including the Mediterranean and the Indian subcontinent. Several countries have developed large programs, which address multiple facets of prevention—patient/provider education, carrier screening, and establishing access to preimplantation and prenatal diagnosis [10]. In Cyprus, for example, couples intending to marry within the Orthodox church are required to obtain a certificate demonstrating that thalassemia screening has been performed [11]. Several states within India provide treatment for affected individuals and efforts for nation-wide comprehensive prevention centers are underway [12]. International attention to hemoglobinopathies and thalassemias is paramount as immigration has increased prevalence of these diseases in areas where they were previously scarce [13]. Recognizing their global importance, the World Health Organization adopted a resolution to improve prevention and management of thalassemias and sickle-cell disease during the 63rd World Health Assembly in 2010 [14].

Fragile X Syndrome is the most common inherited form of developmental disability worldwide, with an estimated prevalence of 1 in 3600 males and 1 in 6000 females across all ethnic groups. The syndrome is X-linked dominant and caused by an unstable expansion of a trinucleotide repeat, with over 200 repeats considered a full mutation. Unlike carrier testing for an autosomal recessive disorder, Fragile X carrier status has important reproductive health implications both for the individual as well as potential risks for offspring [15]. Carriers of 55 to 200 repeats are themselves at risk for Fragile X-associated premature ovarian insufficiency and Fragile X-associated tremor/ataxia syndrome. Currently, both ACOG and ACMG do not recommend universal prenatal carrier screening and instead recommend offering screening to women with a family history of developmental disability, autism, Fragile X-associated conditions, or upon patient request [16,17].

Carrier screening programs for women who do not have any family history suggestive of Fragile X has been debated for a variety of reasons. Proponents of screening cite the severity of the disease and the fact that its incidence is similar to other disorders that are already screened for as rationale for a universal program. Additionally, it has been demonstrated that screening all women prenatally is cost effective when compared to the cost of raising a child with Fragile X [18]. However, Fragile X carrier screening raises distinct counseling challenges. When considering Fragile X screening, a family history must include developmental, neurodegenerative, and reproductive issues across multiple generations. Furthermore, most patients are not aware of Fragile X and the complexity of X-linked inheritance prior to counseling. Lastly, opponents of screening point to the ethical dilemma of potentially labeling a child as a permutation carrier, a status which do not have clinical significance until adulthood [19]. The debate regarding offering all women of reproductive age screening for Fragile X is an important one and will continue to evolve as our understanding of the condition and the implications of screening grows.

## 2. Present

While there have been various successful screening programs developed for CF, TSD, and thalassemias, it is currently not possible to screen for all known disorders within any given population. Currently, the American College of Obstetrics and Gynecology and the American College of Medical Genetics and Genomics have published professional guidelines addressing screening for several different conditions. While these are subject to change, the Table 3 provides a snapshot of existing clinical practice within the United States.

**Table 3:** Summary of currently available ACOG and ACMG screening guidelines.

Screen	ACOG (Year of Publication)	ACMG (Year of Publication)
Cystic Fibrosis	Screening should be offered to all women of reproductive age (2001, reaffirmed 2011) [5]	Screening should be considered by all couples for use for use before conception or prenatally (2001, reaffirmed 2013) [6]
Spinal Muscular Atrophy	Preconception and prenatal screening is not recommended in the general population (2009) [20]	Carrier testing should be offered to all couples regardless of race or ethnicity (2008, reaffirmed 2013) [21]
Fragile X	Population carrier screening is not recommended (2010) [16]	Population carrier screening is not recommended except as part of a well-defined clinical research protocol (2005) [17]
Hemoglobinopathies	Individuals of African, Southeast Asian, and Mediterranean descent are at increased risk for being carriers of hemoglobinopathies and should be offered carrier screening and, if both parents are determined to be carriers, genetic counseling (2007) [3]	Not currently addressed
Ashkenazi Jewish Descent	Individuals of AJ ancestry should be offered screening for four disorders—CF, TSD, Familial Dysautonomia (FD) and Canavan Disease (CD) and should be made aware of the availability of testing for five additional diseases—Fanconi Anemia Group C, Gaucher disease type I, Niemann-Pick disease type A, Bloom syndrome and Mucolipidosis type IV (2009) [22]	Screening should be offered for CF, TSD, Familial Dysautonomia, Canavan Disease, Fanconi anemia (Group C), Niemann-Pick (Type A), Bloom syndrome, Mucolipidosis IV, and Gaucher disease (2008) [23]
Expanded Carrier Screening	Not currently addressed	The proper selection of appropriate disease-causing targets for general population-based carrier screening (*i.e.*, absence of a family history of the disorder) should be developed using clear criteria, rather than simply including as many disorders as possible (2013) [24]

## 3. Future

Technologic advances coupled with increased understanding of the genetic contribution to disease make the move from current standard of practice which involves a gene-by-gene, disorder-by-disorder evaluation of a limited set of mutations to an expanded screen of many genes and many disorders in one time a possibility. Due to wider availability of various technologies including arrays and next-generation sequencing, there has been a push to screen for many different mutations in many different genes in relatively the same time-frame and potentially similar cost as detecting mutations at a single gene. Furthermore, it has been suggested that ancestry-based screening may result in an unequal distribution of genetic testing and will miss these disorders in the populations that are not screened. Also, standard measures to ascertain race and ethnicity may be too restrictive, especially in light of increased admixture or when an individual’s background is unknown [25]. Consequently, relying on self-reported background to guide screening may not be as useful as in the past. As an attempt to address these potential issues, commercial labs have developed expanded screening panels, which are often referred to as “pan-ethnic” or “universal” screening panels.

Expanded panels are comprised of a wide-variety of disorders and are marketed to all individuals, regardless of background [26]. The majority of the disorders screened for are not part of the disorders currently recommended by ACMG or ACOG. The bulk of these panels presently use array-based approaches, though next-generation sequencing techniques are also being utilized. Sequencing has been shown to allow for more comprehensive searches for mutations even within a select panels of diseases [27]. However, clinical experience and information regarding the utility of expanded panels, regardless of technique utilized, is limited and is currently under investigation. While expanded carrier screening may hold a great deal of promise, there are some important challenges posed by the approach.

First, the appropriate composition of expanded panels and their clinical benefits must be clearly defined. There are exome-sequencing based approaches, for example, that promise to screen for hundreds of disorders at a time [27]. Expanded panels have received criticism because some of the disorders selected may not be clinically significant, have an overall low frequency or a variable onset and clinical course [28].

Second, the consent for such panels becomes increasingly complicated as more and more disorders are included. The age of onset and severity of each disorder will differ. A substantial number of individuals screened will be identified as a carrier of at least one disease on a panel, though the chance of both partners being a carrier for one disorder is low. It needs to be clear to patients that the rate of detection of carrier status will vary by disorder and the background of the individual being tested [26]. In addition, next-generation sequencing based approaches are utilized more routinely, how to report and address incidental findings and variants of uncertain significance must be clearly outlined. Presently, ACOG recommends that personalized genome/exome testing only be used as part of clinical trials until prospective studies are available to further define clinical utility [29]. Consent for such expanded panels is time-consuming and can be a challenge in the primary care setting. A survey of obstetricians and gynecologists regarding their opinions of expanded carrier testing revealed that only one-third of subjects felt comfortable providing pre-test counseling and fewer felt comfortable explaining the results of expanded panels. The survey also revealed that the majority of the providers believe that expanded panels should be offered pre-conception as part of family planning [30].

Ascertainment of genetic risks based on family history is a standard component of a preconceptual/prenatal consultation. ACOG requires that obstetric providers ordering tests must educate patients about the implications of genetic testing for the entire family [31]. While there may be potential liability concerns, the primary reason to provide appropriate counseling and screening is to ensure true informed consent and the best possible clinical outcomes.

The clinical benefit of such panels must be balanced against cost, both of the test itself as well as the additional consultations required for proper post-test counseling and clinical follow-up. Clinical follow-up can be lengthy, which poses a special challenge in the prenatal setting where a patient has a limited time-frame to undergo prenatal diagnosis and make family planning decisions. For example, the partner of a patient who is found to be a carrier on an expanded panel may opt for whole-gene sequencing to detect mutations that would not otherwise be detected. Until there is more information about the cost-savings of these tests, they will not be uniformly available to all patient populations.

Expanded panels for carrier screening within the preconceptual/prenatal setting have arrived, along with the challenges and unanswered questions that arise whenever new technologies are implemented and current clinical paradigms are shifting. Recommendations from professional organizations and patient groups will continue to evolve to address these challenges.
